# The assessment of work endurance in disability evaluations across European countries

**DOI:** 10.1371/journal.pone.0202012

**Published:** 2018-09-17

**Authors:** Henk-Jan Boersema, Bert Cornelius, Wout E. L. de Boer, Jac J. L. van der Klink, Sandra Brouwer

**Affiliations:** 1 University of Groningen, University Medical Centre Groningen, Department of Health Sciences, Community and Occupational Medicine, Groningen, The Netherlands; 2 Research Centre for Insurance Medicine AMC-UMCG-UWV-VUMC, Amsterdam, The Netherlands; 3 Swiss Academy of Insurance Medicine, University Hospital Basel, Basel, Switzerland; 4 Tilburg University, Tilburg School of Social and Behavioral Sciences, Tranzo Scientific Center for Care and Welfare, Tilburg, The Netherlands; University of Twente, NETHERLANDS

## Abstract

**Purpose:**

Chronic disease is often associated with a reduced energy level, which limits the capacity to work full-time. This study aims to investigate whether the construct work endurance is part of disability assessment in European countries and what assessment procedures are used. We defined work endurance as the ability to sustain working activities for a number of hours per day and per week.

**Materials and methods:**

We conducted a survey using two self-constructed questionnaires. We addressed 35 experts from 19 countries through the European Union of Medicine in Assurance and Social Security (EUMASS). We gathered descriptive data on various aspects of (the assessment of) work endurance.

**Results:**

Experts from 16 countries responded. In most countries work endurance is assessed. We found few professional guidelines specific for the assessment of work endurance. Both somatic and mental diseases may cause limited work endurance. Methods to assess work endurance vary, objective methods rating as most suitable. Almost half of the countries report controversies on the assessment of work endurance.

**Conclusions:**

Work endurance is recognised and assessed as an aspect of work disability assessment in Europe. However, controversies exist and evidence based guidelines, including reliable and valid methods to assess work endurance, are lacking.

## Introduction

Recent updates of the global burden of disease studies by the World Health Organization show that in the general population chronic diseases account for 76.6% of years lost to disability [1,2]. In the workforce across European countries the proportion of employed persons reporting chronic diseases has increased from 19.3% in 2010 to 20.8% in 2014 [3,4]. In 2011, 19% of persons aged 15–64 years in Europe reported to have some physical and/or mental activity limitation at work, in 38% of cases caused by chronic disease [5].

Apart from specific disease symptoms and health complaints, chronic disease is likely to be associated with reduced physical and mental energy level and activity limitations, eventually limiting work performance in general and the ability to work full-time in particular [3,6,7]. Almost 25% of persons with chronic health problems work part-time compared with 14% of those without disability [8]. On average they work fewer hours than the general population and they more often work part-time compared to healthy workers due to differences in fatigue and emotional exhaustion [9–11].

The International Classification of Functioning, Disability and Health (ICF) is a useful framework to map associations between chronic disease and physical and/or mental activity limitation at work [12]. The ICF defines disability as an umbrella term for impairments in body functions and structures, limitations of activities, and restrictions of participation. Reduced physical and mental energy level are classified in the ICF-domain *Body functions and structures* with the term (impairments in) *Energy level*. Also classified in that domain is the physical ability to sustain activities with the term *General physical* endurance. The construct *Inability to work full time* is not specifically classified in the ICF. In our study, energy deficits include both reduced physical and mental energy levels. This is in accordance to the disability assessment procedures in social security setting, and also to the definition of Philips (2015) [13] i.e. “the psychophysiological condition needed for physical activity or mental processing over time in and out of the actual workplace.”

Reduction of working hours is a frequently applied work accommodation for workers with a chronic disease having difficulty to work full-time, improving the match between work demands and work capacity [14,15]. In a sample of individuals with a chronic disease eligible for a rehabilitation program, the most preferred and realised work accommodations included fewer working hours [16]. In a population of employees with a chronic disease, the need for adjusting working times was reported by 6.2% of all employees, and by 11.0% of those with mental disorders [14]. In a representative sample of workers with various chronic somatic diseases, reduced working hours were most frequently mentioned as work adjustment in 5.8% of cases. In that study 58.8% reported problems at work related to physical endurance and weariness [17]. In a study among working cancer survivors, the most common adjustment was reducing the number of work hours per week [18]. In a review exploring work-related problems in multiple sclerosis, higher disease duration was found to be a determinant of reduction in number of hours worked per week [19].

In the Netherlands, to compensate for income loss, long-term sick listed workers with a limited ability to work due to chronic disease, may apply for disability benefit. The ability to work, including the number of hours per day and per week the claimant is able to work, is evaluated by insurance physicians (IPs) from the Dutch Social Security Institute (SSI). In the Dutch social security system a limitation of working hours due to chronic disease usually results in partial disability. In this paper we introduce the term *Work Endurance*, i.e. the physical and mental ability of a person to sustain working activities in hours per day and hours per week. A professional guideline has been introduced recently to support Dutch IPs in their assessment of the number of hours a claimant is able to work per day and per week [20]. This expert-based guideline includes three indications to consider a claimant’s work endurance as being limited: general energy deficit, reduced availability for work due to medical treatment and prevention of future health deterioration. Despite the availability of this guideline Dutch IPs experience difficulties in assessing possible limitation of working hours among disability benefit claimants, e.g. regarding the number of working hours considered to be normal and whether psychosocial factors should be taken into account [21]. A Dutch study showed that 48% of disability benefit claimants were assessed by IPs using the guideline as having a limitation of working hours and granted partial disability benefit [22]. Another Dutch study among IPs showed large inter-doctor variation in limitation of working hours as disability assessment outcome [23].

In western countries the evaluation of work disability is typically performed by medical examiners who report their findings to social insurance [24,25]. It is known that in different countries different elements are included in the assessment of disability benefits [8,24] and it is unknown if the ability to work a number of hours per day and per week is assessed in all countries. Scientific publications on assessing work endurance in social insurance in European countries and information about whether the assessment of work endurance is part of the assessment of disability benefit are lacking. For international comparison more research about the assessment of work endurance as an important aspect of disability assessment in European countries is warranted [24,26].

We studied if and how in European countries work endurance is assessed as part of the overall disability benefit assessment. Our main question is: “Is work endurance assessed as part of the application of disability benefit?”. If yes: “Are professional evidence-based guidelines for the assessment of work endurance available?”; “What causes are considered to be acceptable for limited work endurance?”; “By which methods is work endurance measured?”; “Do controversies on the assessment of work endurance exist?”

## Materials and methods

### Study setting and participants

We invited experts from 19 European countries: Belgium, Croatia, Czech Republic, Finland, France, Germany, Ireland, Italy, the Netherlands, Norway, Poland, Portugal, Romania, Serbia, Slovakia, Slovenia, Sweden, Switzerland and the United Kingdom (UK). We identified experts through the secretariat of the European Union of Medicine in Assurance and Social Security (EUMASS), a network of national associations of insurance medicine in 19 European countries [27]. EUMASS aims to offer a platform to exchange experiences within the field of insurance medicine between various insurance-related organizations in Europe, mainly focusing on public social security. Each national association is represented in the EUMASS council by up to two staff medical advisor(s), i.e. experts in disability assessment, and may nominate one deputy for each representative. We invited all council members, 35 experts, representing the 19 countries. In the total group of EUMASS expert representatives, the number of eligible respondents per country ranged from one to three. As we invited the total group of 35 eligible EUMASS representatives we were not able to expand the sample by additional members.

### Design and procedures

We invited the participants to fill in two self-constructed surveys consecutively from June 2014 through April 2015. The language of the survey administration was English for all countries. The questionnaire used in the first survey was independently pilot-tested for readability and usability by four practicing insurance physicians and the questionnaire in the second survey by three researchers with expertise in disability assessment. In the first survey experts received a link to a web-based questionnaire with items on the assessment of work endurance. A second questionnaire was sent by email directly to 17 participants in the first survey from 13 countries who had volunteered for the second survey. In both surveys a first and second reminder was sent after four and eight weeks, respectively. Participants from the same country whose answers were not unanimous, were approached separately by email with a request to clarify. Under Dutch law approval of this study by the Medical Ethical Board of the University Medical Centre Groningen was not necessary.

### Measures

In the first survey questionnaire data were gathered on country, profession and expertise of participants. This questionnaire focused on various general aspects of work endurance and its assessment with eight items: the number of working hours per day and per week that is considered normal, the assessment of work endurance as part of the overall disability assessment, the professional assessing work endurance, rules or guidelines that are used, accepted cause(s) for limited work endurance, methods by which work endurance is assessed and any controversies on the assessment of work endurance.

The second survey questionnaire with 12 additional items aimed to provide more detail on work endurance and how it is assessed. It gathered information on the evaluation of the maximum duration to sustain specific activities, the general evaluation of the maximum duration to work in suitable work, specific diseases associated with limited work endurance, causes for limited work endurance and methods suitable to assess work endurance. Suitability was rated on a scale 0–10 (0 = totally unsuitable; 10 = very suitable). Health conditions listed in the second survey questionnaire were grouped according to the International Classification of Disease, 10th edition (ICD-10) [28]. For the first and second questionnaire, see the supplementary S1 Table.

### Data analyses

Data from the first survey round were collected using Unipark software and automatically transferred in SPPS. Data from the second survey round were collected by e-mail and manually added to the SPSS file by the first author (HJB). Data were analysed with IBM SPSS version 22.0 for Windows. Simple frequency statistics and cross tabulations were used. We checked for inconsistencies in respondents in those countries with two or three representatives. If inconsistencies were found, we contacted the representatives and tried to reach consensus. If no consensus could be reached we included the positive answer in the analysis. In those countries with only one representative or respondent it was impossible to check for inconsistencies. If participants filled in a range instead of an absolute number, the mean was taken as value.

## Results

### Participants and response rate

In the first survey data were obtained from 24 of the 35 (response rate 68.6%) potential responders and from 16 of the 19 (84.2%) countries. From seven countries more than one expert responded. Ireland, Portugal, Serbia did not respond. Twenty-four participants filled in the first questionnaire: 13 insurance physicians, six medical advisors, one researcher, one assessment doctor, one medical assessor, one occupational physician and one general practitioner. Eighteen (75%) of these conduct disability assessments in practice. Six were involved in another way, such as medical advice, education, management and organisation and policy making. Seventeen experts from 13 countries were approached in the second survey. Twelve experts (response rate 70.6%) from ten countries (76.9%) responded. From two countries more than one expert responded. Belgium, Finland, Italy, Slovakia, Switzerland and the United Kingdom did not respond. Thus, full data were obtained from 10 countries, provided by 11 participants.

### Number of standard working hours

The range in standard full time working hours per day across countries was from 7.5 (Belgium, Finland, Norway, UK) to 8.3 (Switzerland). The range in standard full time working hours per week was from 35.0 (France) to 42.0 (Switzerland).

### Assessment of work endurance

The assessment of work endurance is part of the disability assessment in 13 of 16 countries. In two of these 13 countries answers to this item were inconsistent. Work endurance is assessed by an insurance or occupational physician. In one country the answer to this item was inconsistent. Formal rules for the assessment of work endurance as part of regulations for work disability assessment in general are used in ten countries. Only in the Netherlands a professional guideline specific for the assessment of work endurance is in use. In four countries the assessment of work endurance includes the evaluation of the maximum duration a person is able to sustain specific activities without interruption, such as walking, standing or sitting. The assessment of work endurance includes the evaluation of the maximum duration a person is able to work in suitable work in five countries. In one country answers were inconsistent on both of these items. For detailed information per country, see Table 1.

**Table 1:** The assessment of work endurance in European countries (n = 16).

	BE	HR	CZ	FI	FR	DE	IT	NO	PL	RO	SK	SL	SE	CH	NL	UK
Assessment of work endurance part of the assessment of work ability	**+**/-	**+**/-	+	-	+	+	+	+	-	+	+	+	+	+	+	-
Assessment of WE by insurance physician	+	+	**+**/-	na	+	+	+	+	na	+	+	+	-	+	+	na
Formal rules and/or guidelines	-	-	+	-	+	+	-	+	+	+	+	+	+	-	+	-
Assessment of WE includes specific activities	mis	-	-	na	-	-	mis	-	na	**+**/-	mis	+	+	mis	+	na
Assessment of WE includes generic evaluation	mis	-	-	na	-	+	mis	+	na	**+**/-	mis	+	+	mis	+	na

+ = yes; - = no; +/- = inconsistent; mis = missing answer; na = not applicable

BE = Belgium; HR = Croatia; CZ = Czech Republic; FI = Finland; FR = France; DE = Germany; IT = Italy; NO = Norway; PL = Poland; RO = Romania; SK = Slovakia; SL = Slovenia; SE = Sweden; CH = Switzerland; NL = Netherlands; UK = United Kingdom

### Causes of limited work endurance

Physical and mental disorders are accepted causes of limited work endurance in all countries. Diseases most mentioned as frequently being associated with limited work endurance are diseases of the musculoskeletal system and connective tissue, mental disorders and diseases of the circulatory system. In seven countries answers to this item were inconsistent (not in table). Psychosocial factors are accepted causes in ten countries, health complaints in eight countries and environmental factors in five countries.

### Indications to limit work endurance

General energy deficit is reported to be an indication to limit work endurance by eight countries. In one country the answer to this item was inconsistent. In six countries reduced availability for work due to medical treatment is an indication to limit work endurance. In seven countries prevention of future health is an indication to limit work endurance. In two countries answers to this item were inconsistent, see Table 2.

**Table 2:** Indications to limit work endurance in European countries (n = 13).

Indications	BE	HR	CZ	FR	DE	NO	PL	RO	SK	SL	SE	NL	UK	Total n
General energy deficit	mis	+	+	-	+	+	mis	**+**/-	mis	+	+	+	mis	8
Reduced availability due to medical treatment	mis	+	-	+	+	-	mis	+	mis	-	+	+	mis	6
Prevention of future health deterioration	mis	+	+	-	+	+	mis	**+**/-	mis	-	**+**/-	+	mis	7
Other aspects	mis	-	+	-	-	+	mis	+	mis	-	+	-	mis	4

+ = yes; - = no; +/- = inconsistent; mis = missing answer

BE = Belgium; HR = Croatia; CZ = Czech Republic; FR = France; DE = Germany; NO = Norway; PL = Poland; RO = Romania; SK = Slovakia; SL = Slovenia

SE = Sweden; NL = Netherlands; UK = United Kingdom

Diseases most mentioned as causes of limited work endurance through general energy deficit are musculoskeletal diseases and mental disorders. Neoplasms and mental disorders are most mentioned as causes of limited work endurance through reduced availability due to medical treatment. Musculoskeletal diseases and mental disorders are most mentioned as causes of limited work endurance through prevention of further health deterioration (not in table).

### Methods to assess work endurance

Clinical test, functional capacity evaluation and psychological test are the most used methods to assess work endurance, see Table 3. Participants from four countries provided inconsistent answers to this item. In all countries different combinations of the listed methods are mentioned as most suitable to assess work endurance.

**Table 3:** Methods (and expert suitability rating: 0–10) used to assess work endurance in European countries (n = 13).

Method	BE	HR	CZ	FR	DE	IT	NO	RO	SK	SL	SE	CH	NL	Mean rating
Semi structured Interview	-	+ (9)	**+**/- (7)	- (5)	- (5)	-	+	- (7)	+	**+**/- (5)	+ (5)	+	+ (8)	6.4
Ergometry	-	- (10)	**+**/- (9)	-	+ (7)	-	+	+ (10)	+	+ (10)	+ (8)	+	- (4)	8.3
Functional Capacity Evaluation	+	- (10)	**+**/- (10)	- (8)	+ (9)	+	+	+ (9)	+	+ (7)	+ (8)	+	- (5)	8.3
Psychological test	-	+ (10)	**+**/- (8)	- (2)	+ (6)	-	+	+ (8.5)	+	+ (8)	+ (8)	+	**+**/- (7)	7.2
Clinical test	-	+ (10)	+ (10)	+ (7)	+ (5)	-	+	+ (8.5)	+	+ (9)	+/- (8)	+	**+**/- (7)	8.1
Test in rehabilitation center	-	+ (9)	- (8)	- (9)	+ (9)	-	+	- (6.5)	-	+ (6)	+/- (8)	+	- (7)	7.8
Self-report questionnaire	-	- (8)	- (5)	- (1)	- (3)	-	+	- (2)	-	+ (3)	+ (5)	+	**+**/- (8)	4.4

+ = yes; - = no; +/- = inconsistent; BE = Belgium; HR = Croatia; CZ = Czech Republic; FI = Finland; FR = France; DE = Germany; IT = Italy; NO = Norway; PL = Poland; RO = Romania; SK = Slovakia; SL = Slovenia; SE = Sweden; CH = Switzerland; NL = Netherlands; UK = United Kingdom

Clinical tests include flexibility tests of joints, cardiovascular and respiratory functional diagnostics, functional capacity evaluation, ergometry, clinical examination, visual field test, imaging like X-ray, MRI and ultrasound, electromyography, endoscopy, laboratory test, audiometry and electro-encephalography. Other tests include tests on cognitive function, psychological tests, semi-structured interviews, self-report questionnaire and psychiatric evaluation.

Ergometry and functional capacity evaluation rate highest with both 8.3 points (on a scale 0–10) as being the most suitable method to assess work endurance, see Table 3. Semi-structured interview and self-report questionnaire rate lowest with 6.4 and 4.4 points respectively.

### Controversies on the assessment of work endurance

Controversies are reported on the assessment of work endurance in 10 countries. Nine of these countries provided short descriptions of controversies, see Table 4.

**Table 4:** Controversies on the assessment of work endurance in European countries (n = 9).

Country	Description of controversy ^a^
Belgium	In fact there is no debate at all about that topic! More and more accents on reintegration measures.
Croatia	Such a controversy is basically a consequence of nonexistence of formal rules and professional guidelines for the assessment of work endurance in Croatia.
Norway	It is discussed if partial sick leave during the sickness absence period has beneficial effects on the duration of sick leave, and how beneficial it is for patient and employer.
Romania	At present, the approach is considered to be too medical; the current difficult socio-economic conditions make very difficult an appropriate socio-professional evaluation (missing the possibilities of intervention, agencies, etc.).
Slovakia	Controversy between findings and information from patients.
Slovenia	There should be possibility for oldest people to choose working part time—for example 6 or 4 hours not only 8 hours.
Sweden	The latest test (AFU) is still a pilot project to be reported to the department. The reference system, representing the demands of the job market, has been criticized by the unions.
Switzerland	Diverging opinions as to what is a legitimate reason to be off work, both in politics and in law enforcement as in the medical profession. Different schools of sick leave & any doctor can write somebody off work.
Netherlands	Claims are much higher and more frequent then would be expected, especially in litigation. Other restrictions versus restricted work endurance: outcome can be different.

^a^ Descriptions are verbatim; only obvious spelling mistakes are corrected.

## Discussion

Our results show that work endurance is assessed as part of the overall disability assessment in a majority of countries. Work endurance is considered to be normal, if a person is able to work full-time, ranging from 35 to 42 hours per week across countries. Limited work endurance can be described as the inability to work full time. In almost all cases work endurance is conducted by a medical examiner specialised in insurance medicine. In all countries both physical and mental disorders are accepted causes of limited work endurance. Most mentioned accepted causes are musculoskeletal diseases, mental disorders and diseases of the circulatory system. Health complaints, psychosocial and environmental factors are additionally accepted as causes of limited work endurance in some countries. In most countries indications are given to limit work endurance, general energy deficit being the most frequent. Methods to assess work endurance vary considerably across countries, objective methods rating highest. Use of expert-based professional guidelines specific for the assessment of work endurance is very limited and evidence-based guidelines do not exist at all. On items as to whether work endurance is assessed at all, causes of limited work endurance, indications to limit work endurance and methods to assess work endurance, some participants from the same country gave inconsistent answers. In almost half of countries controversies on the assessment of work endurance exist.

The definition of work endurance we introduced in this paper, is confirmed by our results, showing that work endurance can be described as the physical and mental ability of a person to sustain working activities in hours per day and hours per week. Some countries seem to view work endurance from a broad perspective, including both medical and psychosocial factors. By doing so, they seem to adopt a biopsychosocial perspective as outlined in the ICF [12]. Although social security institutes in most western countries have developed new assessment procedures based on the ICF [8], the ICF is not yet a generally accepted framework to describe human functioning in disability assessment [12,29]. Use of the ICF may potentially support the assessment of work endurance by providing a point of reference for the ability of a person to work over a certain period of time. Although limited work endurance is an important aspect of work disability, in the ICF it is not specifically defined. The ICF includes only related concepts on the level of functioning, i.e. “general physical endurance” and “energy level”, respectively defined as “functions related to the general level of tolerance of physical exercise or stamina”, and as “mental functions that produce vigour and stamina” [8].

From the investigated countries it is reported that musculoskeletal diseases, mental disorders and diseases of the circulatory system are the most prevalent accepted causes of limited work endurance. These chronic diseases range among the most prevalent conditions where work adjustments as to working times are needed and implemented [15]. This indicates that these categories of chronic diseases are broadly recognized as being importantly associated with limited work endurance.

A variety of methods is used to assess functional limitations including work endurance, such as clinical interview, physical examination, functional capacity evaluation, self-report questionnaire, expert assessment by medical specialists. None of these methods have proven reliability and validity [23].

This study shows that a guideline on assessing work endurance is used only in the Netherlands. In general, guidelines for the evaluation of work disability are scarce, do not meet sufficient quality levels and are not evidence-based [30]. The indications for limited work endurance included in the Dutch guideline and confirmed by some other countries, especially general energy deficit, are not based on scientific evidence. Lack of evidence-based guidelines will cause variability across assessors [23,31,32].

### Strengths and limitations

To our best knowledge, the present study is the first to examine work endurance and its assessment in disability settings in different countries. This study provides information which can facilitate understanding of similarities and differences in the assessment of work endurance across a number of European countries. The participants were contacted through the EUMASS network and may therefore be considered to be experts in the field.

Our study has limitations as well. In the total group, of the number of potential respondents per country differed from one to three. The group of expert representatives did not change during the study period, making it impossible to look for inconsistencies when only one respondent from a country responded. We checked for inconsistencies in respondents in those countries with two or three representatives. If inconsistencies were found, we contacted the representatives and tried to reach consensus. If no consensus could be reached we included the positive answer in the analysis. We were not able to expand to other experts from the same country to discuss inconsistencies due to the chosen sampling method. In the first survey, 24 of the 35 potential responders reacted, from which 17 agreed to participate in the second survey. Of them, 12 responded in the second survey. Whether respondents and non-respondents differ in sociodemographics, cultural aspects and/or how it may have influenced their responses on the survey could not be examined, because we and/or EUMASS did not have this information available. This may restrict the generalisability of our results. We have insufficient reliable data to assess whether non-response has caused selection bias. It is an exploratory description of opinions of experts, not allowing any statements about the practice in these countries.

From several countries more than one participant responded. Some answers of participants were not unanimous, even after they were specifically requested to clarify. Given the descriptive character of our study we deemed it relevant to report on these inconsistent answers instead of merely concluding that apparently policy on items concerned is absent. This lack of uniformity may be the result of the way in which the questions were formulated, but seem more likely to result from differences among experts. This is in line with the findings of a recent systematic review showing that medical evaluations of work disability in general show high variability and often low reliability [33]. The inconsistencies of answers may also be illustrations of controversies on work endurance, other than those that were reported on. Our study does not inform on differences and similarities between countries on aspects of work endurance that may arise from different regulations regarding assessment of work ability, including work endurance.

### Recommendations for future research

In many disability evaluations the assessment of work endurance is an issue. Reliable and valid instruments and methods to assess work endurance seem not to be in practice. Research could focus on the prevalence of limited capacity to work full time and on methods to establish this limitation in individuals. If reliable and valid instruments and methods to assess work endurance are not available, further research is needed to develop them. Such research is best conducted among chronically-ill workers, with repeated measurements of energy levels over time in and out of the actual workplace. Methods able to assess work endurance with sufficient reliability and validity should then be tested for feasibility, i.e. whether they can be implemented in practice of insurance physicians assessing disability benefit claims. If so, they can eventually be included in an evidence-based guideline for the assessment of work endurance.

## Conclusion

Notwithstanding existing controversies and inconsistent answers from some countries, across European countries it is broadly recognised that limited work endurance has impact on work ability of chronically-ill workers applying for disability benefit. We conclude that the assessment of the ability to work full time is an issue in a majority of European countries. However, methods to assess work endurance vary and evidence-based guidelines are lacking. More research is needed to develop reliable and valid instruments and methods to assess work endurance of disability benefit claimants with chronic diseases.

## Supporting information

S1 Table Items in questionnaire on work endurance for experts. (DOCX)
