# Supplementary material for: The assessment of work endurance in disability evaluations across European countries
Source: PLoS One. 2018 Sep 17;13(9):e0202012. doi: 10.1371/journal.pone.0202012 (PMC6141062; doi:10.1371/journal.pone.0202012)
Supplement: S1 Table — (DOCX) [file pone.0202012.s001.docx]

**Supplementary Table S1:** items in questionnaire on work endurance for experts (provided options and explanations in italics).

**Items questionnaire in first round**

1. What is generally seen as a normal number of working hours per day in your country?
2. What is generally seen as a normal number of working hours per week in your country?
3. Is the assessment of work endurance part of the assessment of work disability (short or long term) in your country?
4. Who assesses work endurance in your country?

*provided options: insurance physician, occupational physician, general practitioner, medical specialist, labour expert, case manager, rehabilitation specialist, other:*

1. Are there any formal rules and/or professional guidelines for the assessment of work endurance in your country? Could you please provide us with these rules and/or professional guidelines and/or give references below?
2. What is an accepted cause for restricted work endurance in the context of work disability assessment in your country?

*provided options: physical disorders, mental disorders, health complaints (pain, fatigue), environmental factors (e.g. workplace, region), psychosocial factors (distress, anxiety, coping behavior), risk factors (life style, obesity, physical inactivity), other:*

1. Is any of the following methods used regularly for work endurance assessment in your country? *provided options: semi-structured interview, ergometric test, functional capacity evaluation, psychological test, clinical test, assessment in rehabilitation center, self-report questionnaire, other:*
2. Is there any controversy on the assessment of work endurance in your country? Would you be so kind as to summarize this controversy below?

**Items questionnaire in** **second round**

*In general the assessment of work endurance may include the evaluation of different time-related aspects, such as the maximum duration (in minutes or hours) a person is able to sustain specific activities without interruption, e.g. walking, standing up or sitting down, and/or generically the maximum duration (in hours per day/week) a person is able to work in suitable work.*

1. Does the assessment of work endurance in your country include the evaluation of the maximum duration a person is able to sustain specific activities without interruption?
2. Does the assessment of work endurance in your country include the generic evaluation of the maximum duration a person is able to work in suitable work?

*Limited work endurance may be associated with a variety of (sub)chronic diseases. For some diseases this association may be strong, while for some others it may be weaker.*

1. Is limited work endurance restricted or associated with specific diseases in the allocation of work endurance in your country? If yes, could you please indicate below three specific diseases you as a professional think are most frequently associated with limited work endurance?

*In the Netherlands, insurance physicians use a guideline for the assessment of work endurance. Across specific diseases, according to this guideline, work endurance may be limited due to a general energy deficit or a reduced availability for work due to medical treatment. The guideline states that work endurance may also be limited in order to prevent future health deterioration. Please indicate below whether these aspects are reasons to limit work endurance in your country.*

1. Can the aspect general energy deficit be a reason to limit work endurance in your country? Please indicate below three specific diseases you think are the most frequently associated with energy deficit.
2. Can the aspect reduced availability for work due to medical treatment be a reason to limit work endurance in your country? Please indicate below three situations you think are the most frequently associated with reduced availability for work due to medical treatment.
3. Can the aspect prevention of future health deterioration be a reason to limit work endurance in your country? Please indicate below three specific diseases you think are most frequently associated with prevention of future health deterioration.
4. Can other aspects (besides energy deficit, reduced availability and prevention) be reasons to limit work endurance in your country? If yes, please indicate these aspects below.

*Respondents in the first round of this survey indicated various methods commonly used in their countries to assess work endurance. These are (in random order: semi structured interview, ergometric test, functional capacity evaluation, psychological test, clinical test, assessment in rehabilitation center and self-report questionnaire.*

1. Please rate each of these methods below by giving a number (0: not suitable -10: very suitable) as to indicate the extent to which in your professional opinion the method is suitable to assess work endurance.

*Provided options: semi structured interview, ergometric, functional capacity evaluation, psychological test, clinical test, assessment in rehabilitation center, self-report questionnaire.*

*The assessment of work endurance could possibly be improved by combining two or more of the above mentioned methods.*

1. Please indicate below which combinations of two (or more) of the above mentioned methods in your professional opinion are the most suitable to assess work endurance.

*In the first round questionnaire two methods were most frequently cited: clinical test and psychological test. Both tests may include different elements.*

1. Is the clinical test used in the assessment of work endurance in your country? If yes please indicate below which elements are included in this test.
2. Is the psychological test used in the assessment of work endurance in your country? If yes, please indicate below which elements are included in this test.

*Any of the above mentioned methods may have specific indications as to disease, function or activity.*

1. In your professional opinion, do any of the above methods to assess work endurance have specific indications? If yes, please explicate the indication(s) where applicable.
